# Gastrointestinal symptoms, inflammation and hypoalbuminemia in chronic kidney disease patients: a cross-sectional study

**DOI:** 10.1186/s12882-015-0209-z

**Published:** 2015-12-11

**Authors:** Xuehan Zhang, Nisha Bansal, Alan S. Go, Chi-yuan Hsu

**Affiliations:** Department of Health Care, Peking Union Medical College Hospital, Chinese Academy of Medical Sciences, Beijing, China; Division of Nephrology, University of California-San Francisco, San Francisco, CA USA; Division of Nephrology, University of Washington, Seattle, WA USA; Division of Research, Kaiser Permanente Northern California, Oakland, CA USA

**Keywords:** Chronic kidney disease, Gastrointestinal symptoms, Hypoalbuminemia

## Abstract

**Background:**

Few studies have focused on investigating hypoalbuminemia in patients during earlier stages of chronic kidney disease (CKD). In particular, little is known about the role of gastrointestinal (GI) symptoms. Our goal in this paper is to study how GI symptoms relate to serum albumin levels in CKD, especially in the context of and compared with inflammation.

**Methods:**

We performed a cross-sectional study of 3599 patients with chronic kidney disease enrolled in the Chronic Renal Insufficiency Cohort (CRIC) study. All subjects were asked to complete the Modification of Diet in Renal Disease (MDRD) study patient symptom form. Our main predictor is GI symptom score. Serum level of C-reactive protein (CRP) was measured as well. Main outcome measures are serum albumin levels and prevalence of hypoalbuminemia.

**Results:**

Of the participants assessed, mean serum albumin was 3.95 ± 0.46 g/dL; 12.7 % had hypoalbuminemia. Patients with lower estimated glomerular filtration rate (eGFR) were likely to have more GI symptoms (apparent at an eGFR <45 ml/min/1.73 m^2^). Patients with worse GI symptoms had lower dietary protein intake. GI symptoms, like inflammation, were risk factors for lower serum albumin levels. However, adding GI symptom score or CRP into the multivariable regression analysis, did not attenuate the association between lower eGFR and lower albumin or hypoalbuminemia.

**Conclusions:**

Increased prevalence of GI symptoms become apparent among CKD patients at relatively high eGFR levels (45 ml/min/1.73 m^2^), long before ESRD. Patients with more severe GI symptoms scores are more likely to have hypoalbuminemia. But our data do not support GI symptoms/decreased protein intake or inflammation as being the main determinants of serum albumin level in CKD patients.

**Electronic supplementary material:**

The online version of this article (doi:10.1186/s12882-015-0209-z) contains supplementary material, which is available to authorized users.

## Background

Hypoalbuminemia is well known to be an important problem in patients with end-stage renal disease (ESRD), where it is one of the strongest predictors for death and other adverse outcomes [1–3]. Low serum albumin levels are also an adverse prognostic sign in non-kidney disease patients [4–6]. However, few studies have focused on investigating hypoalbuminemia in patients during earlier stages of chronic kidney disease (CKD) [7–9]. In particular, little is known about the role of gastrointestinal (GI) symptoms.

Prior studies of malnutrition in ESRD have emphasized the prevalence of anorexia and resultant reduced oral intake. Few studies however, have examined GI symptoms in patients with milder degrees of renal dysfunction. One paper, from the Modification of Diet in Renal Disease (MDRD) study, reported that the prevalence of “uremic” symptoms such as nausea, vomiting and anorexia actually emerge at higher glomerular filtration rate (GFR) levels than previously realized (i.e. these were not just present in ESRD patients) [10].

Prior studies, especially in the ESRD literature, have emphasized that serum albumin concentration correlates not only with lower protein intake, but also with inflammation, the severity of which can be assessed using a metric such as level of C-reactive protein (CRP), an acute phase reactant [11–13]. We therefore undertook the current study to better understand the potential contributions of GI symptoms and inflammation on the association of kidney function and serum albumin levels in CKD.

## Methods

### Study population

This was a cross-sectional analysis of baseline data from the Chronic Renal Insufficiency Cohort (CRIC) study. CRIC is a multi-center observational study of 3,939 adults aged 21 to 74 years old at baseline with mild to moderate CKD with estimated GFR (eGFR) ranging from 20–70 ml/min/1.73 m^2^. The eGFR was estimated using the MDRD equation. Participants were recruited from seven clinical centers (13 recruitment sites) throughout the United States from 2003 through 2008. The study design, methods, baseline characteristics, the inclusion and exclusion criteria have been previously described [14, 15]. The study was approved by the local institutional review boards at the CRIC data coordinating center (University of Pennsylvania) and recruitment sites (University of Pennsylvania, John Hopkins University, University of Maryland, University Hospitals of Cleveland Case Medical Center, MetroHealth Medical Center, Cleveland Clinical Foundation, St. John’s Health System, Wayne State University, University of Michigan at Ann Arbor, University of Illinois at Chicago, Tulane University, Kaiser Permanente of Northern California and University of California, San Francisco). All CRIC participants gave informed written consent for the collection of information for the purpose of research.

All CRIC participants had symptoms assessed at baseline using the MDRD study Patient Symptom Form [10] described below. No specific dietary instructions were given to enrollees by study investigators. After excluding (overlapping) CRIC participants who did not complete the Patient Symptom Form (*n* = 22) or had missing serum albumin/CRP (*n* = 65), or missing 24-hour urine urea, urine protein, urine albumin quantification (*n* = 263) or missing height and weight information (*n* = 10), our final study size was 3,599 (see Additional file 1: Figure S1).

### Assessment of GI symptoms

The MDRD Patient Symptom Form includes 23 items covering a range of symptoms which may be related to kidney disease, such as muscle cramps or itching. Patients were asked about the number of days in the past month that they had each of the symptoms listed. If a symptom was present, patients rated the severity of the symptom on a scale of 1 (mild, symptoms did not interfere with usual activities), 2 (moderate, symptoms interfered somewhat with usual activities) or 3 (severe, symptoms were so bothersome that usual activities could not be performed). For the current analysis we *a priori* decided to focus on five of the GI symptoms which we judged to be more likely to be associated with dietary intake, including “A bad taste in your mouth,” “Loss of appetite,” “Nausea or being sick to your stomach,” “Vomiting,” and “Abdominal bloating or gas.” To create a severity score for each symptom, we multiplied the number of symptom days by the severity scale (i.e. higher score represented more pronounced symptoms). We found that all four scores were higher among patients with lower eGFR except for “Abdominal bloating or gas”. So our final “GI symptom score” was the summation of the individual severity scores for “A bad taste in your mouth”, “Loss of appetite”, “Nausea or being sick to your stomach”, “Vomiting”. We classified *a priori* participants without symptoms as the reference group for all comparisons and then divided the remaining participants into tertiles to avoid assumptions of linear effect. (In sensitivity analysis we did include “Abdominal bloating or gas” also into the GI symptom score.)

### Measurement of serum albumin

Serum albumin level was measured by dye-binding assay (Ortho Clinical Diagnostics). We defined hypoalbuminemia as serum albumin < 3.5 g/dL [16, 17].

### Assessment of other covariates

High sensitive CRP was measured by end-point nephelometry (Siemens BN™ II System) [18]. Patients were divided into three categories: CRP < 3.00 mg/L, CRP between 3.00 and 10.00 mg/L, and CRP ≥ 10.00 mg/L [7, 19–21].

At the baseline CRIC visit, urine urea nitrogen, total protein and albumin excretion were determined from a 24-hour urine collection. Dietary protein intake was estimated according to the following formula [22]: dietary protein intake (g/24 h) = 6.25 × [24 h urine urea nitrogen + 0.031 × body weight] + 24 h urine protein. Dietary protein intake was normalized to body weight [23, 24]. Urine urea nitrogen concentration was determined by endpoint spectrophotometric assay (Ortho Clinical Diagnostics). Total urine protein concentration was determined with the turbidometric method with benzthonium chloride (Roche Diagnostics) and total urine albumin was done by immunoturbidometric assay (Roche Diagnostics). The samples were rejected and re-collection attempted if total urine volumes were below 500 ml or collection times below 22 h or more than 26 h.

### Statistical analysis

We first examined the distribution of baseline characteristics according to categories of eGFR. Continuous variables were described as mean ± standard deviation or median (25th-75th percentiles) where appropriate and analyzed using analysis of variance. Categorical variables were described using proportions and analyzed using the *χ*^2^ test. CRP was log transformed given the skewed distribution.

**Fig. 1:**
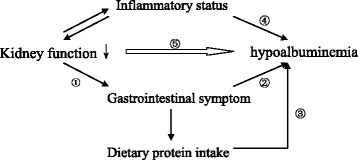
Relationship between reduced kidney function, serum albumin levels, GI symptoms, dietary protein intake and inflammation

We then proceeded to undertake a series of analyses to better understand the relationship between reduced kidney function, GI symptoms, protein intake, serum albumin levels, and other factors which may affect serum albumin levels such as inflammation (please see Fig. 1 for conceptual diagram). Logistic regression analysis was used to estimate odds of presence of specified patient GI symptoms (yes/no) as a function of baseline eGFR, controlling for age, gender, race/ethnicity, diabetes diagnosis and using patients with eGFR ≥60 ml/min/1.73 m^2^ as the reference group (pathway ①). We then determined using linear regression whether worse GI symptoms was correlated with lower serum albumin (and in logistic regression determined whether worse GI symptoms was correlated with higher risk of hypoalbuminemia) (pathway ②). We then adjusted for dietary protein intake to determine whether poor protein intake mediated the association between worse GI symptoms and lower serum albumin levels (pathway ③). In parallel, we examined whether higher CRP was correlated with lower serum albumin (pathway ④).

Finally, we evaluated whether worse GI symptoms and higher CRP explained why patients with lower eGFR had lower serum albumin or whether other mechanisms were important (pathway ⑤). We did this by seeing if the association between lower eGFR and serum albumin levels was attenuated or extinguished by adjusting for GI symptoms, CRP and other factors which may influence serum albumin level such as 24-hour urine albumin excretion.

All statistical analyses were conducted using IBM SPSS Statistics 20.0 (IBM Corporation, Armonk, NY, USA). A *p*-value < 0.05 was considered statistically significant.

### Regulatory approval

De-identified data for this analysis was retrieved from the National Institutes of Diabetes and Digestive and Kidney Disease (NIDDK) Data Repository (https://www.niddkrepository.org/home/) after appropriate institutional review board approval was obtained (University of California San Francisco Committee on Human Research IRB Number: 10–04231).

## Results

The baseline characteristics of the study population are shown in Table 1. Of the 3599 participants in our study, the mean age was 58.4 ± 10.9 years, 55 % were men, the mean BMI was 32.2 ± 7.8 kg/m^2^, mean serum albumin was 3.95 ± 0.46 g/dL; 12.7 % had hypoalbuminemia.

**Table 1:** Characteristics of study population by level of eGFR (ml/min/1.73 m^2^)

		eGFR level (ml/min/1.73 m^2^)				
Characteristic	Total (*N* = 3599)	<30 (*N* = 688)	30–45 (*N* = 1375)	45–60 (*N* = 1166)	≥60 (*N* = 370)	*p* value
Mean age, years, mean (SD)	58.4 ± 10.9	58.8 ± 11.2	59.8 ± 10.6	58.6 ± 10.3	51.6 ± 11.0	<0.001
Men (%)	55.0	47.8	53.5	60.4	57.0	<0.001
Race/Ethnicity						<0.001
White/Caucasian (%)	43.3	37.6	43.2	47.6	40.8	
Black/African American (%)	41.4	40.3	40.4	41.0	48.9	
Hispanic (%)	11.4	18.3	12.9	7.2	5.9	
Other (%)	3.9	3.8	3.5	4.2	4.3	
Diabetes mellitus (%)	48.1	54.2	54.0	42.1	33.2	<0.001
Myocardial Infarcation/Prior Revasculization (%)	22.2	24.7	24.2	21.4	13.0	<0.001
Peripheral Vascular Disease (%)	6.7	11.0	7.1	4.9	2.7	<0.001
Stroke (%)	9.7	10.6	10.7	9.9	4.1	0.001
BMI, kg/m^2^, mean (SD)	32.2 ± 7.8	32.1 ± 8.3	32.5 ± 7.7	32.1 ± 7.9	31.4 ± 7.1	0.1
Serum creatinine, mg/dL, mean (SD)	1.74 ± 0.57	2.56 ± 0.56	1.78 ± 0.32	1.39 ± 0.22	1.14 ± 0.20	
eGFR, ml/min/1.73 m^2^, mean (SD)	42.9 ± 13.4	24.9 ± 3.51	37.8 ± 4.22	51.6 ± 4.23	67.5 ± 7.55	
Albumin level, g/dL, mean (SD)	3.95 ± 0.46	3.86 ± 0.47	3.90 ± 0.47	4.01 ± 0.44	4.07 ± 0.40	<0.001
Hypoalbuminemia (%)	12.7	16.6	15.6	9.0	6.2	<0.001
Dietary Protein Intake, g/kg/day, mean (SD)	0.81 ± 0.31	0.78 ± 0.31	0.80 ± 0.30	0.83 ± 0.30	0.87 ± 0.35	<0.001
CRP, mg/L, median (IQR)	2.59 (1.07, 6.33)	2.83 (1.08, 6.97)	2.90 (1.20, 6.71)	2.38 (1.00, 6.23)	1.85 (0.89, 4.07)	<0.001
Urine albumin, g/24 h, median (IQR)	0.06 (0.01, 0.55)	0.34 (0.05, 1.52)	0.10 (0.02, 0.71)	0.03 (0.01, 0.21)	0.01 (0.01, 0.08)	<0.001
4-variable^a^ GI symptom score, mean (SD) median (range)	11.1 ± 25.9 0(0,348)	14.0 ± 27.9 2(0, 250)	12.2 ± 27.1 0(0,348)	8.7 ± 24.1 0(0,282)	9.0 ± 22.2 0(0,150)	<0.001

^a^the 4 variables are “A bad taste in your mouth?”, “Loss of appetite?”, “Nausea or being sick to your stomach?”, “Vomiting?”

Conversion factors for units: Serum creatinine in mg/dL to μmol/L, ×88.4

**Fig. 2:**
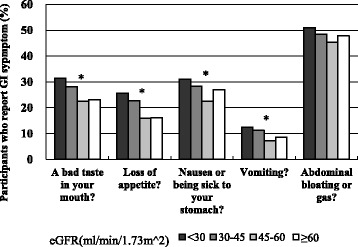
Prevalence of GI symptoms across eGFR categories. The * identifies *P* < 0.01 for a chi-square test among the groups with different eGFR values

Characteristics of those who did and did not have GI symptoms are compared in the Table 2. In general, those with lower eGFR indeed were more likely to have GI symptoms (Fig. 2). They also had lower serum albumin and higher CRP levels (Table 1).

**Table 2:** Characteristics of study population with and without GI symptoms

Characteristics	Without GI symptoms (*N* = 1897)	With GI symptoms (*N* = 1702)	*p* value
Mean age, years, mean (SD)	59.3 ± 10.7	57.3 ± 11.0	<0.001
Men (%)	61.1	48.2	<0.001
Race/Ethnicity			
White/Caucasian (%)	48.0	38.1	<0.001
Black/African American (%)	38.4	44.8	
Hispanic (%)	9.1	13.9	
Other (%)	4.5	3.1	
Diabetes mellitus (%)	44.2	52.4	<0.001
Myocardial Infarcation/Prior Revasc (%)	21.4	23.5	0.082
Peripheral Vascular Disease (%)	5.0	8.6	<0.001
Stroke (%)	7.9	11.8	<0.001
BMI, kg/m^2^, mean (SD)	31.8 ± 7.3	32.6 ± 8.4	0.002
Serum creatinine, mg/dL, mean (SD)	1.69 ± 0.52	1.79 ± 0.62	<0.001
eGFR, ml/min/1.73 m^2^, mean (SD)	44.2 ± 13.0	41.4 ± 13.7	<0.001
Albumin level, g/dL, mean (SD)	3.99 ± 0.44	3.90 ± 0.47	<0.001
Hypoalbuminemia (%)	10.2	15.5	<0.001
Dietary Protein Intake, g/kg/day, mean (SD)	0.84 ± 0.32	0.77 ± 0.29	<0.001
CRP, mg/L, median (IQR)	2.41 (1.01, 5.68)	2.82 (1.13, 7.08)	0.003
Urine albumin, g/24 h, median (IQR)	0.05 (0.01, 0.50)	0.09 (0.01, 0.64)	<0.001

As shown in Table 3, lower eGFR was an independent risk factor for having more severe GI symptoms even after controlling for demographics and presence or absence of diabetes mellitus (pathway ①).

**Table 3:** Odds Ratios for Specific GI symptoms (pathway ①) Adjusted for age, sex, race/ethnicity, diabetes

Symptom	Odds Ratio (95 % CI)			
	eGFR ≥60 ml /min/1.73 m^2^ (*n* = 370)	eGFR 45–60 ml /min/1.73 m^2^ (*n* = 1166)	eGFR 30–45 ml /min/1.73 m^2^ (*n* = 1375)	eGFR < 30 ml /min/1.73 m^2^ (*n* = 688)
A bad taste in your mouth?	1.0 (reference)	0.99(0.75–1.32) *P* = 0.9	1.22(0.92–1.62) *P* = 0.2	1.34(0.99–1.82) *P* = 0.06
Loss of appetite?	1.0 (reference)	1.21(0.88–1.68) *P* = 0.2	1.83(1.34–2.52) *P* < 0.001	2.01(1.43–2.82) *P* < 0.001
Nausea or being sick to your stomach?	1.0 (reference)	0.95(0.72–1.25) *P* = 0.7	1.24(0.95–1.63) *P* = 0.1	1.32(0.98–1.78) *P* = 0.06
Vomiting?	1.0 (reference)	1.08(0.70–1.67) *P* = 0.7	1.67(1.11–2.54) *P* = 0.02	1.76(1.13–2.75) *P* = 0.01

Patients with worse GI symptoms had lower serum albumin concentrations and higher risk of hypoalbuminemia in univariate regression (Table 4) (pathway ②). GI symptoms was negatively correlated with dietary protein intake (r = −0.141, *P* < 0.01). And dietary protein intake was positively correlated with serum albumin level (r = 0.094, *P* < 0.01) (pathway ③). By adding dietary protein intake into the regression model, we found that the association between GI symptoms and low albumin was slightly weakened but not abolished (Table 4) (pathway ② + ③).

**Table 4:** How serum albumin levels and prevalence of hypoalbuminemia vary by GI symptoms (Pathway ② + ③)

	Outcomes			
	Absolute change in serum albumin g/dL (95 % CI)		Hypoalbuminemia odds ratio (95 % CI)	
Predictor GI symptom score	Unadjusted	Adjusted for dietary protein intake	Unadjusted	Adjusted for dietary protein intake
None (score of 0) (*n* = 1897)	reference	reference	1.0 reference	1.0 reference
Low tertile (score of 1–5) (*n* = 608)	−0.07(−0.11 ~ −0.03) *p* = 0.002	−0.06(−0.10 ~ −0.02) *p* = 0.004	1.44(1.09 ~ 1.89) *p* = 0.01	1.41(1.07 ~ 1.85) *p* = 0.02
Middle tertile (score of 6–24) (*n* = 585)	−0.09(−0.13 ~ −0.04) *p* < 0.001	−0.08(−0.12 ~ −0.04) *p* < 0.001	1.67(1.28 ~ 2.18) *p* < 0.001	1.63(1.25 ~ 2.13) *p* < 0.001
High tertile (score of 25–360) (*n* = 509)	−0.13(−0.18 ~ −0.09) *p* < 0.001	−0.12(−0.16 ~ −0.08) *p* < 0.001	1.77(1.34 ~ 2.33) *p* < 0.001	1.71(1.29 ~ 2.25) *p* < 0.001

Patients with higher CRP levels had lower serum albumin concentrations. Specifically, compared with participants with CRP <3.00 mg/L (*n* = 1965), those with CRP 3.00–10.00 mg/L (*n* = 1200) had serum albumin levels which were 0.05 g/dL (*p* = 0.003) lower and those with CRP ≥10.00 mg/L (*n* = 434) had serum albumin levels which were 0.13 g/dL (*p* < 0.001) lower (pathway ④).

To examine how much GI symptoms and inflammation mediated the association between lower eGFR and hypoalbuminemia, we simultaneously controlled for GI symptoms and CRP in a model with eGFR as the exposure and serum albumin as the outcome. As shown in Tables 5 and 6, in unadjusted analyses, patients with lower eGFR had lower serum albumin levels and higher risk of hypoalbuminemia. After adding GI symptom score or CRP into the regression analysis, neither factor attenuated the association between lower eGFR and lower albumin or hypoalbuminemia (pathway ⑤). But we found that the association between eGFR and low albumin was greatly weakened by adding 24-hour urine albumin excretion into the regression model. Finally, in multivariable analysis, lower eGFR was an independent risk factor for lower serum albumin and higher risk of hypoalbuminemia after controlling for demographics and presence or absence of diabetes mellitus. But after adding GI symptom score, CRP and 24-hour urine albumin excretion into the fully adjusted model, the association between lower eGFR and lower serum albumin/higher risk of hypoalbuminemia was basically abolished.

**Table 5:** How serum albumin levels vary by eGFR (pathway ⑤)

	Outcomes					
	Absolute change in serum albumin, g/dL (95 % CI)					
Predictor eGFR (ml/min/1.73 m^2^)	Unadjusted	Adjusted for GI symptom score	Adjusted for CRP	Adjusted for 24-hr albuminuria	Multivariable adjusted^a^	Fully adjusted^b^
≥60(*n* = 370)	reference	reference	reference	reference	reference	reference
45–60 (*n* = 1166)	−0.06 (−0.11 ~ −0.003) *p* = 0.04	−0.06 (−0.11 ~ −0.003) *p* = 0.04	−0.05 (−0.10 ~ 0.002) *p* = 0.06	−0.03 (−0.08 ~ 0.02) *p* = 0.2	−0.08 (−0.13 ~ −0.03) *p* = 0.002	−0.03 (−0.07 ~ 0.02) *p* = 0.2
30–44 (*n* = 1375)	−0.16 (−0.22 ~ −0.11) *p* < 0.001	−0.16 (−0.21 ~ −0.11) *p* < 0.001	−0.15 (−0.21 ~ −0.10) *p* < 0.001	−0.09 (−0.14 ~ −0.05) *p* < 0.001	−0.16 (−0.21 ~ −0.11) *p* < 0.001	−0.07 (−0.11 ~ −0.02) *p* = 0.005
<30 (*n* = 688)	−0.21 (−0.27 ~ −0.15) *p* < 0.001	−0.20 (−0.26 ~ −0.14) *p* < 0.001	−0.20 (−0.25 ~ −0.14) *p* < 0.001	−0.08 (−0.13 ~ −0.03) *p* = 0.003	−0.19 (−0.24 ~ −0.13) *p* < 0.001	−0.04 (−0.09 ~ 0.01) *p* = 0.1

^a^Adjusted for age, sex, race/ethnicity, diabetes

^b^Adjusted for age, sex, race/ethnicity, diabetes, CRP, GI symptom score, 24-hour albuminuria

**Table 6:** How prevalence of hypoalbuminemia varies by eGFR (pathway ⑤)

	Outcomes					
	Hypoalbuminemia, Odds Ratio (95 % CI)					
Predictor eGFR (ml/min/1.73 m^2^)	Unadjusted	Adjusted for GI symptom score	Adjusted for CRP	Adjusted for 24-hr albuminuria	Multivariable adjusted^a^	Fully adjusted^b^
≥60 (*n* = 370)	1.0 reference	1.0 reference	1.0 reference	1.0 reference	1.0 reference	1.0 reference
45–60 (*n* = 1166)	1.49 (0.94 ~ 2.38) *p* = 0.09	1.50 (0.94 ~ 2.39) *p* = 0.09	1.46 (0.92 ~ 2.34) *p* = 0.1	1.24 (0.76 ~ 2.02) *p* = 0.4	1.79 (1.11 ~ 2.91) *p* = 0.02	1.30 (0.78 ~ 2.14) *p* = 0.3
30–44 (*n* = 1375)	2.78 (1.78 ~ 4.35) *p* < 0.001	2.73 (1.75 ~ 4.27) *p* < 0.001	2.68 (1.72 ~ 4.19) *p* < 0.001	1.86 (1.16 ~ 2.97) *p* = 0.01	2.98 (1.87 ~ 4.75) *p* < 0.001	1.71 (1.05 ~ 2.78) *p* = 0.03
<30 (*n* = 688)	3.00 (1.88 ~ 4.78) *p* < 0.001	2.91 (1.82 ~ 4.65) *p* < 0.001	2.82 (1.77 ~ 4.51) *p* < 0.001	1.36 (0.82 ~ 2.25) *p* = 0.2	2.90 (1.78 ~ 4.72) *p* < 0.001	1.18 (0.70 ~ 2.00) *p* = 0.5

^a^Adjusted for age, sex, race/ethnicity, diabetes

^b^Adjusted for age, sex, race/ethnicity, diabetes, CRP, GI symptom score, 24-hour albuminuria

In sensitivity analyses, our results were similar when we analyzed the sum of 5 rather than 4 GI symptoms (data not shown).

## Discussion

In summary, in this large cross-sectional study of CKD patients, those with lower eGFR had a higher burden of GI symptoms and there was an association between burden of GI symptoms and lower serum albumin levels. Patients with reduced eGFR also had higher CRP levels which was associated with lower serum albumin levels. But the reason for the hypoalbuminemia in CKD patients does not appear to be due to GI symptoms/decreased protein intake or inflammation.

A diminished appetite and nausea/vomiting are classic “uremic symptoms”. However, few studies have systematically assessed these GI symptoms among CKD patients not on dialysis. One prior study noted that, long before ESRD, starting at stage 3b CKD (eGFR <45 ml/min/1.73 m^2^), GI symptoms such as loss of appetite and nausea/vomiting start to emerge [10]. We are able to extend these observations to a more diverse cohort than the MDRD study participants who were all clinical trial enrollees and had to exceed certain nutritional threshold in order to be allowed to be randomized into a diet manipulation study. Our results suggest that physicians should be aware of the possible onset of GI symptoms relatively early in the course of CKD and take this into account in the course of patient care.

Our study advances our understanding of the clinical implications of these GI symptoms. GI symptoms are risk factors for lower serum albumin levels in CKD patients. It is easy and inexpensive to ask patients about GI symptoms repeatedly over time. Spontaneous restriction of protein intake is known to occur in patients as CKD progresses [25, 26]. Patients with worse GI symptoms had lower dietary protein intake, and it seems plausible that this association was causal (although this cannot be proven in a cross-sectional observational study). Interestingly, controlling for dietary protein intake only partly attenuated the association between GI symptoms and hypoalbuminemia, arguing that this is not that important a pathway connecting the two, although this conclusion should be tampered by the recognition that there is significant measurement error in both the assessment of symptoms and the quantification of dietary protein intake. Further studies are therefore needed to better our understanding of the association between GI symptoms and hypoalbuminemia.

In parallel with our examination of GI symptoms and for comparison, we also examined the association of CRP with serum albumin. CRP is the most frequently measured inflammatory marker and is associated with an increased risk of cardiovascular disease and mortality in both the general population [27–29] and in CKD and ESRD patients [11, 12, 30]. Our studies are consistent with others studies enrolling participants in the range of stage 2–4 CKD which showed that CRP levels are higher at lower levels of GFR [31, 32]. They differ from a report from MDRD which found no relationship between levels of CRP and GFR in univariate analysis [33]. Possibilities for the discrepancy may include the fact that MDRD was a clinical trial which enrolled relatively healthy patients, for example excluding those with serum albumin <3.0 g/dL or body weight <80 % standard body weight [34]. Also, few patients with DM were enrolled [34].

When we controlled for GI symptoms and CRP, we did not attenuate the association between lower eGFR and lower serum albumin levels. Of note, the R^2^ for the model linking dietary protein intake and CRP to serum albumin are relatively low (both 0.01). This differs from the situation in ESRD in which normalized protein catabolic rate (nPCR) and CRP explained more of the variation in serum albumin (R^2^ = 0.13) [12]. An important reason may be that CRP levels are only mildly increased in CKD, not that much higher than the general population [33]. In contrast, CRP levels are more elevated in ESRD. For example, Kaysen et al. concluded that the cross-sectional association of albumin levels with CRP levels is much stronger when CRP level reach >13 mg/L [30].

But in our final analysis, neither GI symptoms nor inflammation appeared to explain why patients with CKD at higher risk for hypoalbuminemia. We found in contrast that 24-hour urine albumin excretion appears to be more important since by additionally adjusting for this, we observed that the association between eGFR and lower serum albumin level was basically abolished. This is consistent with the result of prior cross-sectional analyses [35, 36] and supports the hypothesis that urinary albumin loss are in the causal pathway linking lower eGFR and lower serum albumin levels, although it should be noted that absolute levels of 24-hour urine albumin are quite low in CRIC participants.

Our analysis had several strengths. There are few studies which have systemically collected data on GI symptoms in CKD patients and we were able to relate this to hypoalbuminemia, an important clinical parameter. Our study population was relatively large with a wide range of eGFR and included a high proportion of black and diabetic patients, representative of the U.S. CKD population. GI symptoms were systematically captured using a uniform research protocol. Dietary protein intake was objectively measured using 24-hour urine and not inferred from recall which may be subject to biases.

The present study also has several limitations. First, only one 24-hour urine sample was available to calculate dietary protein intake from urinary urea nitrogen. Second, we use CRP alone to fully adjust for the effects of inflammation. Other acute phase proteins with longer half-lives, such as fibrinogen, α1 acid glycoprotein, may provide additional information if available. We may also be able to develop a more comprehensive understanding of the inflammation-intake paradigm by consideration of cytokines (for example, IL-1, IL-6, TNF-α) or negative acute phase proteins (for example, prealbumin and transferrin) in future studies. Third, the present is a cross-sectional study which limited inferences regarding direction of effect. Fourth, there was no international representation and the high BMI observed may be particular to the American CKD population. Finally, CRIC excluded patients with polycystic kidney disease, multiple myeloma and glomerular diseases on active immunosuppression [14, 15]. So our results may not be generalizable to those patients.

## Conclusions

In conclusion, increased prevalence of GI symptoms become apparent among CKD patients at relatively high eGFR levels, long before ESRD. GI symptoms, like inflammation, are risk factors for lower serum albumin levels. But the presence of GI symptoms (and inflammation) cannot fully explain the prevalence of hypoalbuminemia in CKD patients. More studies are required to better understand the relationship between reduced kidney function and hypoalbuminemia.

## Additional file

Additional file 1: Figure S1. Flowchart used to define the study cohort. (DOC 44 kb)

## Abbreviations

CKD: Chronic kidney disease
CRIC: Chronic Renal Insufficiency Cohort
CRP: C-reactive protein
ESRD: End-stage renal disease
GI: Gastrointestinal
MDRD: Modification of Diet in Renal Disease
nPCR: Normalized protein catabolic rate
