# Supplementary material for: Gastrointestinal symptoms, inflammation and hypoalbuminemia in chronic kidney disease patients: a cross-sectional study
Source: BMC Nephrol. 2015 Dec 11;16:211. doi: 10.1186/s12882-015-0209-z (PMC4676825; doi:10.1186/s12882-015-0209-z)
Supplement: Additional file 1: Figure S1. — Flowchart used to define the study cohort. (DOC 44 kb) [file 12882_2015_209_MOESM1_ESM.doc]

Total CRIC participants = 3939

*340 excluded* due to missing data (overlapping)

*22 excluded* due to not complete the Patient Symptom Form

*65 excluded* due to missing serum albumin/CRP data

*263 excluded* due to missing 24-hour urine urea, urine protein and urine albumin quantification

*10 excluded* due to missing height and weight information

Final N = 3599

**Additional file 1: Figure S1.** Flowchart used to define the study cohort
